# Supplementary material for: Long-Term Environmental Hypoxia Exposure and Haematopoietic Prolyl Hydroxylase-1 Deletion Do Not Impact Experimental Crohn’s Like Ileitis
Source: Biology (Basel). 2021 Sep 8;10(9):887. doi: 10.3390/biology10090887 (PMC8464968; doi:10.3390/biology10090887)
Supplement: Supplementary file 1 [file biology-10-00887-s001.zip › biology-1345824-supplementary.pdf]

biology

MDPI

Supplementary Material

Long-Term Environmental Hypoxia Exposure and Haematopoietic Prolyl Hydroxylase-1 Deletion Do Not Impact Experimental Crohn's Like Ileitis

Cara De Galan[1-3], Martine De Vos[1,2], Pieter Hindryckx[1,4°], Debby Laukens[1-3°] and Sophie Van Welden[1-3°*]

[1] Department of Internal Medicine and Paediatrics, Ghent University, 9000 Ghent, Belgium; cara.degalan@ugent.be (C.D.G.); martine.devos@ugent.be (M.D.V.); pieter.hindryckx@uzgent.be (P.H.); debby.laukens@ugent.be (D.L.)
[2] Ghent Gut Inflammation Group (GGIG), Ghent University, 9000 Ghent, Belgium
[3] VIB Centre for Inflammation Research, 9000 Ghent, Belgium
[4] Department of Gastroenterology, Ghent University Hospital, 9000 Ghent, Belgium
* Correspondence: sophie.vanwelden@ugent.be; Tel.: +32-9-332-58-30
† Joint senior authorship.

SUPPLEMENTARY RESULTS

Genotyping and confirmation of Phd1-deletion in Phd1$^{f/+}$Vav:cre and Phd1$^{f/f}$Vav:cre mice

To confirm Phd1-deletion in the distal ileum samples, PCR reactions were performed for the presence of cre (CRE PCR) and the floxed Phd1 allele (Phd1 cKO PCR) as was initially done at the time of weaning and after sacrification. Genotyping on the distal ileal sections demonstrated that all mice included in the experiments had the expected genotype corresponding with the original genotype (Figure S10). As the presence of cre recombinase in combination with the presence of the floxed allele does not provide information on the degree of recombination, qPCR primers were designed as previously described [1]. Briefly, to detect the Phd1 allele as it occurs in the WT mice, the forward primer binds the Phd1 cDNA sequence at exon 2, before the floxed exons (exon 3 - exon 4), whereas the reverse primer binds exon 3, which will be deleted if recombination occurs (Figure S11, left). As a positive control for effective recombination (as it occurs in all floxed cre-mice), primers were designed in which one binds at the transition between exon 2 and exon 5, which does not occur in WT mice, and the reverse primer binds exon 5 (Figure S11, right). Thus, the allele detected by the first primer pair will be referred to hereafter as "Phd1 wt", whereas the "remaining" Phd1 allele, detected by the second primer pair, will be denoted "Phd1 ko". The primer sequences are provided in Table S1.

When analyzing Phd1 wt allele expression in homozygous Phd1 floxed mice, we observed that Phd1 wt allele expression was not down-regulated in floxed cre mice (Figure S10c). This is however not surprising since we isolated the RNA from an entire ileal piece, containing many other Phd1-positive cells in addition to the Phd1-deleted immune cells and which can therefore explain the lack of a clear decrease in Phd1 wt expression. However, all floxed cre containing mice showed a strong upregulation of the Phd1 ko allele. This is much easier to detect since this Phd1 ko allele is only present in Phd1-deleted immune cells and not at all in non-immune cells. These results confirm effective recombination of the Phd1 allele in distal ileum sections. Literature also confirms the high specificity and efficiency of the vav-promotor. It has been reported that when crossed with floxed mice, Vav:cre mice generate a nearly complete recombination of the floxed gene in all cells of the haematopoeitic lineage [2,3].

SUPPLEMENTARY FIGURES

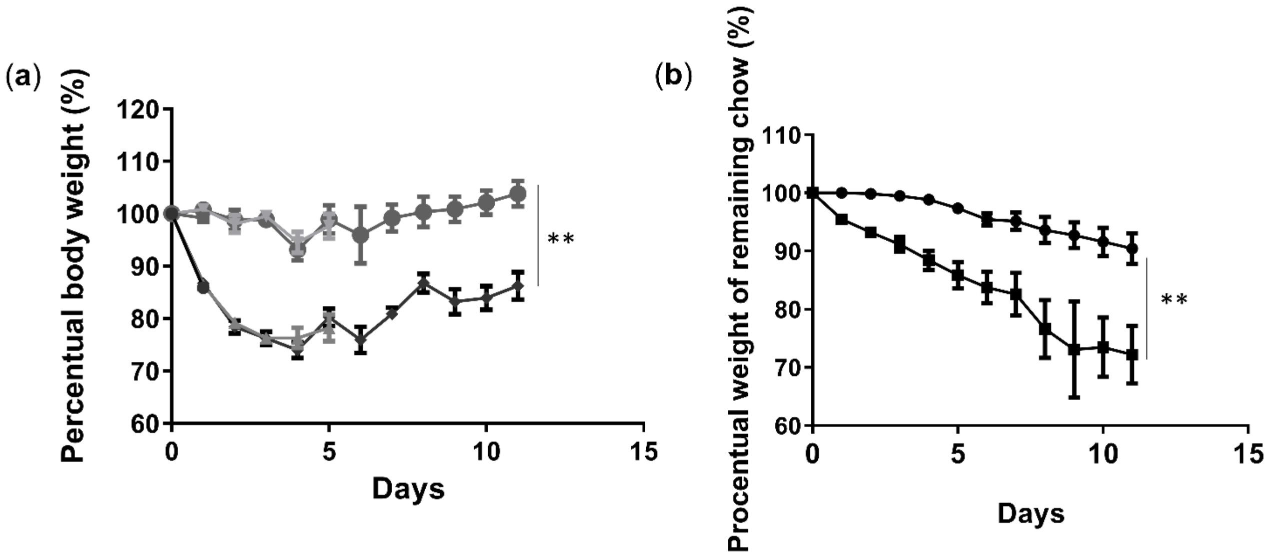

Figure S1. The environmental hypoxia acclimatization period in WT mice is associated with a drop in body weight, due to reduced food uptake. (**a**) Percentual body weight evolution of WT littermates housed in hypoxia for 24 hours ( ), 5 days ( ) and 11 days ( ) and WT littermates housed in normoxia for 24 hours ( ), 5 days ( ) and 11 days ( ). (**b**) Percentual weight follow-up of the remaining food in the cages housed in hypoxia ( ) and normoxia ( ). **p<0.01. Data are represented as the mean ± standard deviation (SD).

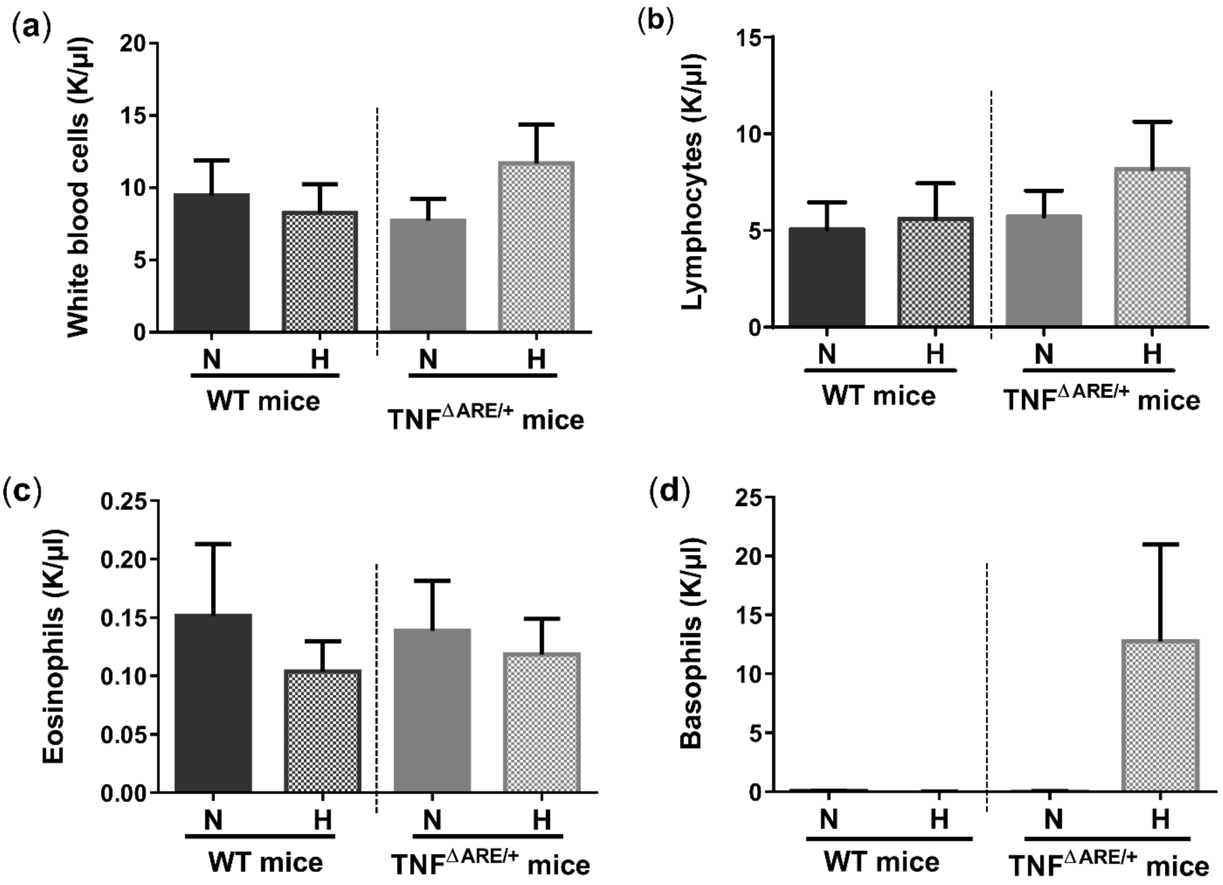

Figure S2. The number of circulatory monocytes and neutrophils are higher in TNF$^{\Delta ARE/+}$ mice housed in hypoxia, but not of other immune cells. The number of circulatory (**a**) white blood cells, (**b**) lymphocytes, (**c**) eosinophils and (**d**) basophils was evaluated. Data are represented as the mean ± SEM. N: Normoxia; H: Hypoxia.

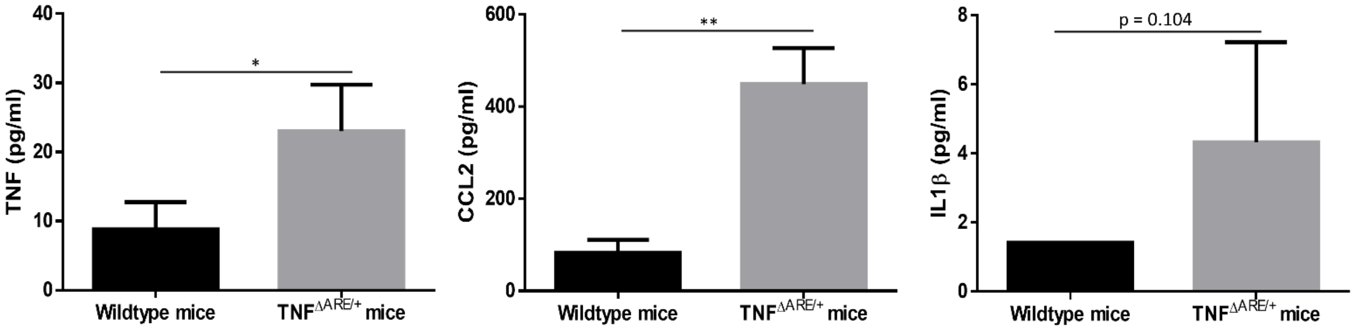

Figure S3. Circulatory proinflammatory cytokines are significantly increased in TNF$^{\Delta ARE/+}$ mice compared with WT littermates in normoxia. Serum levels of TNF, CCL2 and IL1β in TNF$^{\Delta ARE/+}$ mice and WT littermates in normoxia. *$p<0.05$, **$p<0.01$. Data are represented as the mean ± SEM.

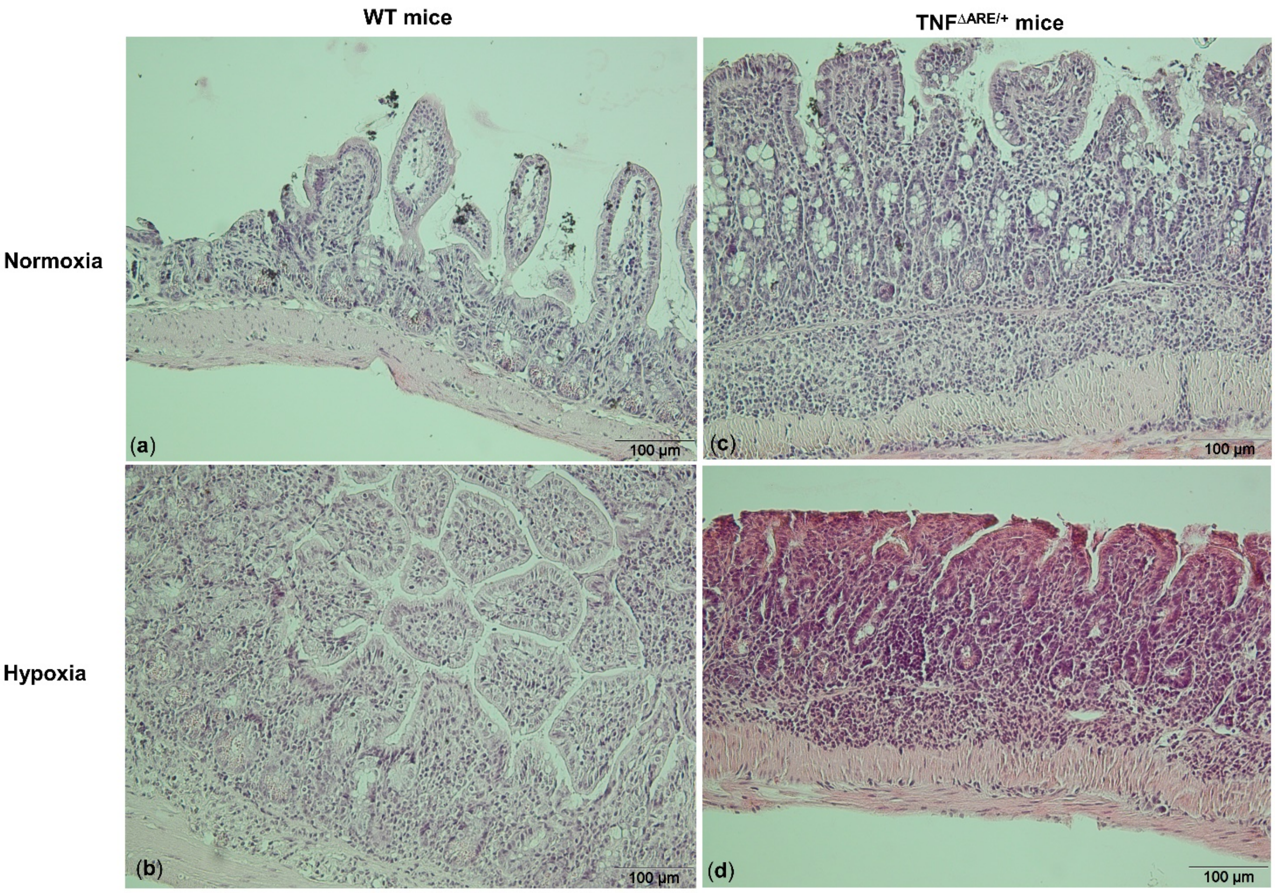

Figure S4. Long-term environmental hypoxia exposure does not influence ileitis development on a histological level. Representative images from H&E sections of the distal ileum from (**a**) a WT mouse housed in normoxia; (**b**) a WT mouse housed in hypoxia; (**c**) a TNF$^{\Delta are/+}$ mouse housed in normoxia; (**d**) a TNF$^{\Delta are/+}$ mouse housed in hypoxia.

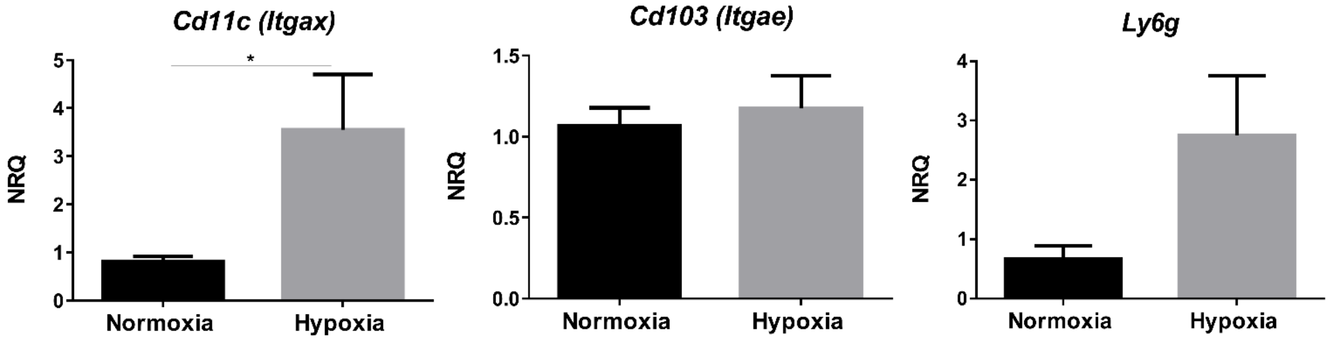

Figure S5. Long-term environmental hypoxia exposure increases the number of mononuclear phagocytes in WT mice. mRNA expression levels of Cd11c (Itgax), Cd103 (Itgae) and Ly6g in the distal ileum of WT. *$p<0.05$. Data are represented as the mean ± SEM. NRQ: Normalized relative quantities.

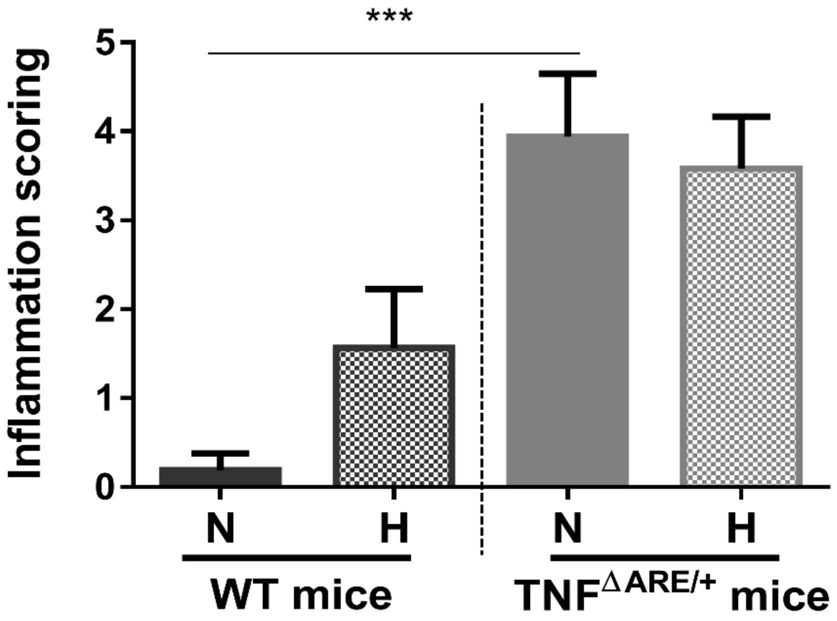

Figure S6. TNF$^{\Delta ARE/+}$ mice housed in normoxia have outspoken signs of ileal inflammation. Total histological inflammation score in WT littermates and TNF$^{\Delta ARE/+}$ mice housed in normoxia and hypoxia. ***$p<0.001$. Data are represented as the mean ± SEM. N: Normoxia; H: Hypoxia.

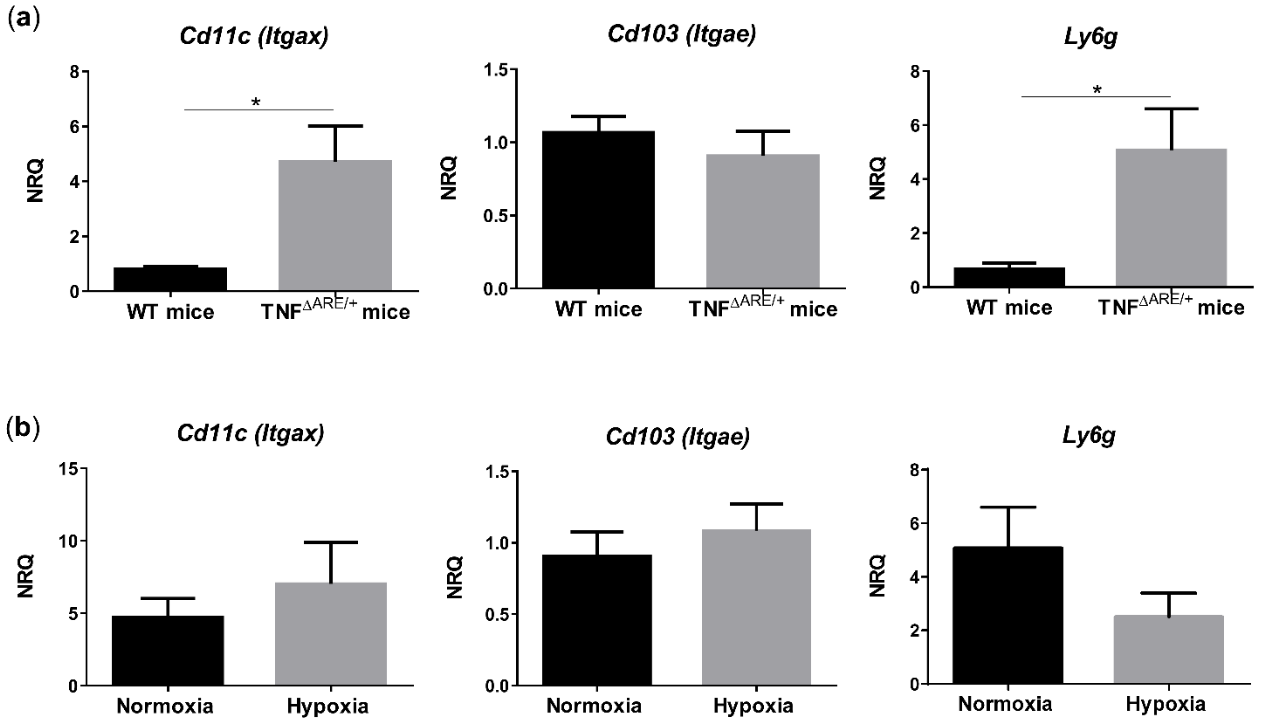

Figure S7. Mononuclear phagocytes are only increased in normoxic TNF$^{\Delta ARE/+}$ mice. (**a**) mRNA expression levels of Cd11c (Itgax), Cd103 (Itgae) and Ly6g in the distal ileum of normoxic WT and TNF$^{\Delta ARE/+}$ mice. (**b**) mRNA expression levels of Cd11c (Itgax), Cd103 (Itgae) and Ly6g in the distal ileum of TNF$^{\Delta ARE/+}$ mice housed in normoxia and hypoxia. *$p<0.05$. Data are represented as the mean ± SEM. NRQ: Normalized relative quantities.

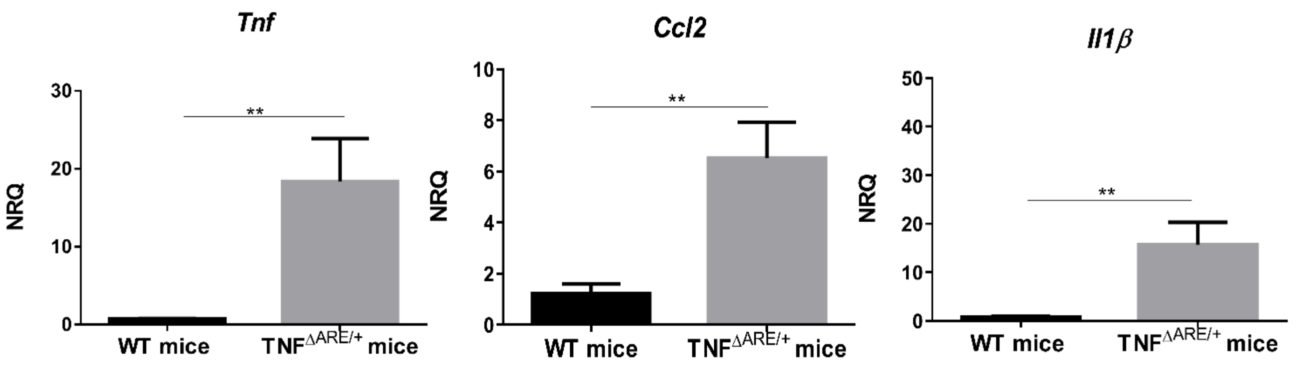

Figure S8. Proinflammatory genes are up-regulated in TNF$^{\Delta ARE/+}$ mice compared to WT littermates housed in normoxia. mRNA expression levels of the proinflammatory genes Tnf, Ccl2 and Il1β in TNF$^{\Delta ARE/+}$ mice and WT littermates housed in normoxia. **$p<0.01$. Data are represented as the mean ± SEM. NRQ: Normalized relative quantities.

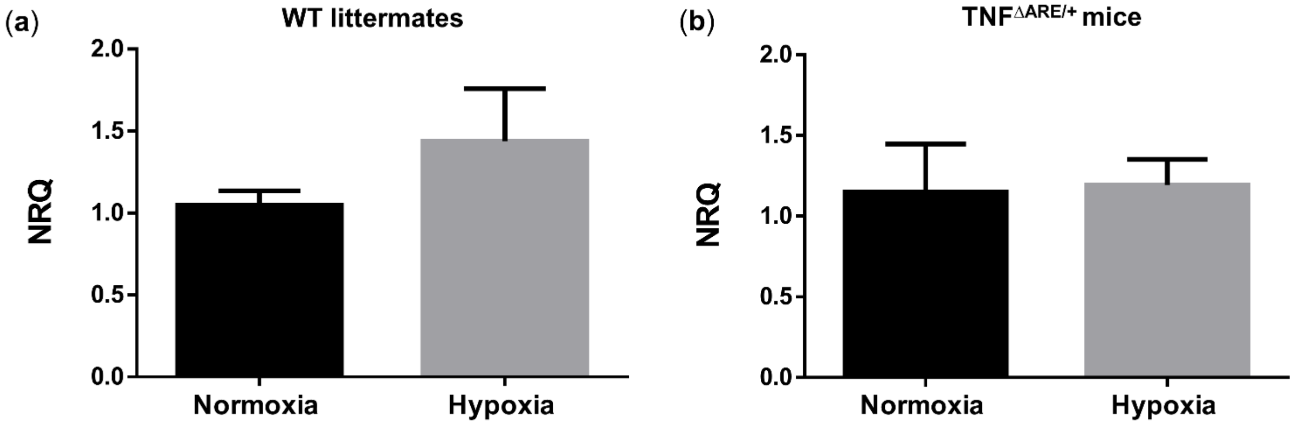

Figure S9. Long-term environmental hypoxia does not alter Phd1 gene expression in the distal ileum of WT and TNF$^{\Delta ARE/+}$ mice. mRNA expression levels of Phd1 in (**a**) wildtype littermates and (**b**) TNF$^{\Delta ARE/+}$ mice housed in hypoxia or normoxia. Data are represented as the mean ± SEM. NRQ: Normalized relative quantities.

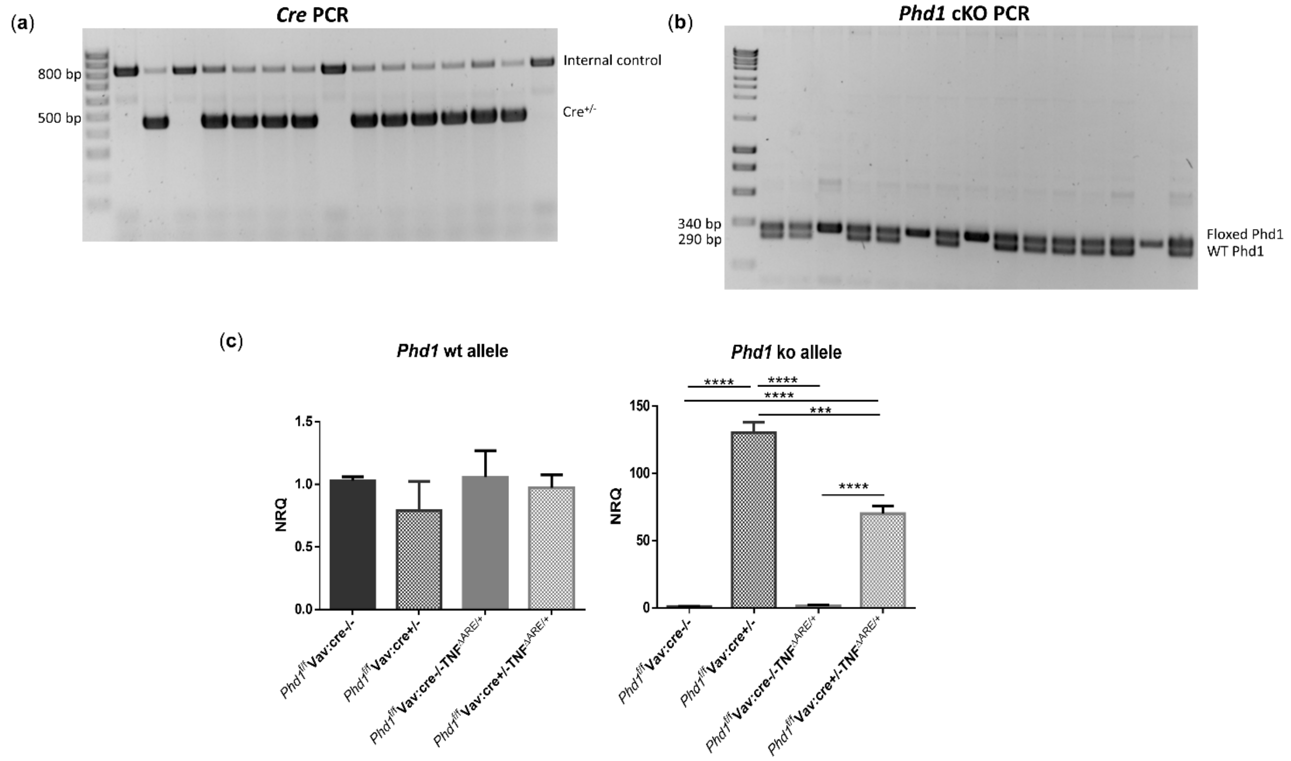

Figure S10. Genotyping of Phd1 deleted $TNF^{\Delta ARE/+}$ mice on DNA extracted from distal ileum. Representative agarose-gels of (**a**) Cre and (**b**) Phd1 conditional knock-out (cko) PCR results from $Phd1^{f/+}$ $Vav{:}cre^{-/-}$, $Phd1^{f/f}$ $Vav{:}cre^{-/-}$, $Phd1^{f/+}$ $Vav{:}cre^{+/-}$, $Phd1^{f/f}$ $Vav{:}cre^{+/-}$, $Phd1^{f/+}$ $Vav{:}cre^{-/-}$ $TNF^{\Delta ARE/+}$, $Phd1^{f/f}$ $Vav{:}cre^{-/-}$ $TNF^{\Delta ARE/+}$, $Phd1^{f/+}$ $Vav{:}cre^{+/-}$ $TNF^{\Delta ARE/+}$ and $Phd1^{f/f}$ $Vav{:}cre^{+/-}$ $TNF^{\Delta ARE/+}$ mice. (**c**) mRNA expression levels of the Phd1 wildtype (wt) and Phd1 knock out (ko) allele in the distal ileum of $Phd1^{f/f}$ $Vav{:}cre^{-/-}$, $Phd1^{f/f}$ $Vav{:}cre^{+/-}$, $Phd1^{f/f}$ $Vav{:}cre^{+/-}$ $TNF^{\Delta ARE/+}$ and $Phd1^{f/f}$ $Vav{:}cre^{+/-}$ $TNF^{\Delta ARE/+}$ mice. ***$p<0.001$ and ****$p<0.0001$. Data are represented as the mean ± SEM. NRQ: Normalized relative quantities.

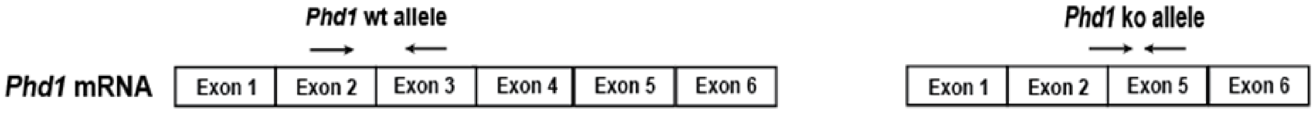

Figure S11. Schematic overview of the binding sites of the 2 qPCR primer pairs to distinguish Phd1 expression in the WT and Phd1$^{f/f}$Vav:cre mice. (**Left**) Phd1 mRNA sequence with the forward primer binding exon 2 and the reverse primer binding exon 3, thereby amplifying the Phd1 allele as it occurs in the WT mice and is denoted as the "Phd1 wt allele". (**Right**) Phd1 mRNA sequence as it occurs in the Phd1$^{f/f}$Vav:cre mice with the forward primer binding at the transition of exon 2 and exon 5, whereas the reverse primer binds exon 5. The resulting amplicon is the Phd1 allele as it occurs in the Phd1$^{f/f}$Vav:cre mice and is denoted as the "Phd1 ko allele".

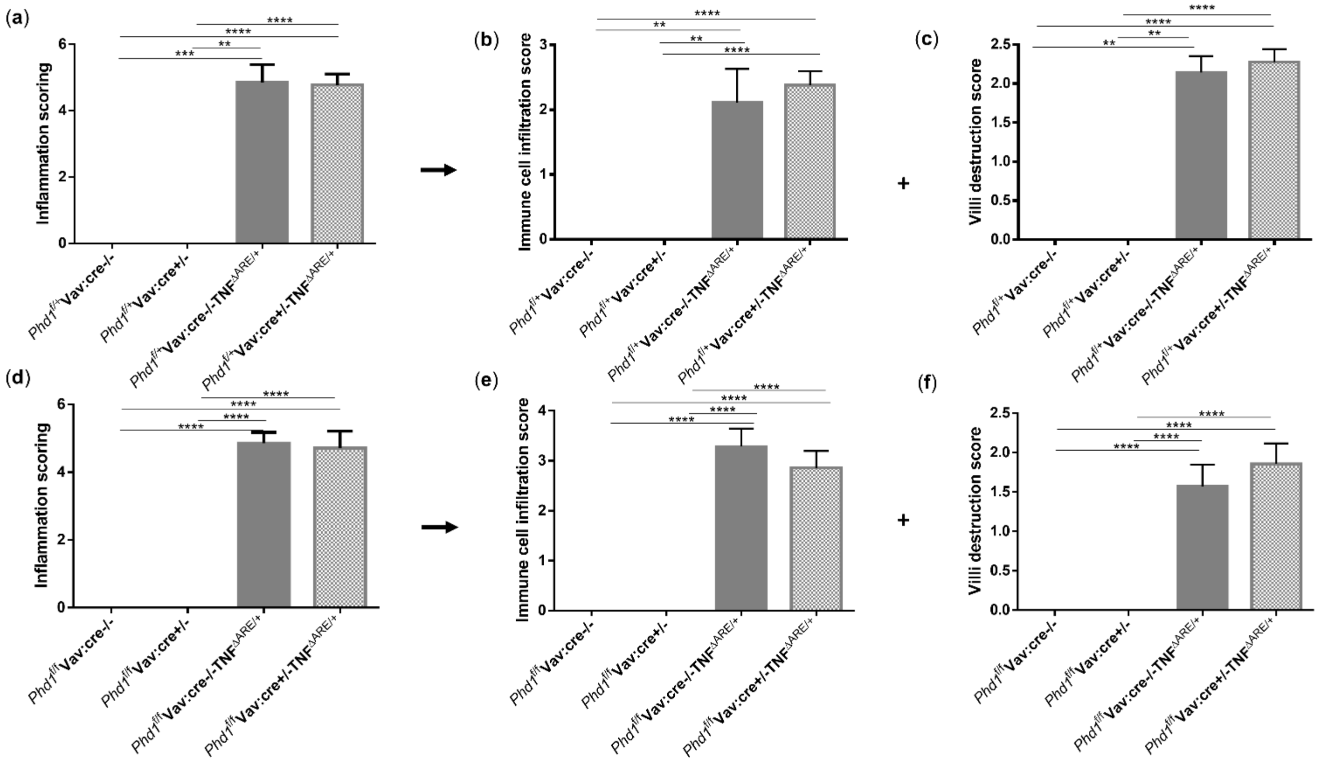

Figure S12. Heterozygous and homozygous Phd1-deletion does not impact ileitis development. (**a**) Total histological inflammation score in Phd1$^{f/+}$Vav:cre$^{-/-}$, Phd1$^{f/+}$Vav:cre$^{+/-}$, Phd1$^{f/+}$Vav:cre$^{-/-}$TNF$^{\Delta ARE/+}$, Phd1$^{f/+}$Vav:cre$^{+/-}$TNF$^{\Delta ARE/+}$, comprising the subscores: (**b**) immune cell infiltration and (**c**) villi destruction. (**d**) Total histological inflammation score in Phd1$^{f/f}$Vav:cre$^{-/-}$, Phd1$^{f/f}$Vav:cre$^{+/-}$, Phd1$^{f/f}$Vav:cre$^{-/-}$TNF$^{\Delta ARE/+}$, Phd1$^{f/f}$Vav:cre$^{+/-}$TNF$^{\Delta ARE/+}$, comprising the subscores: (**e**) immune cell infiltration and (**f**) villi destruction.

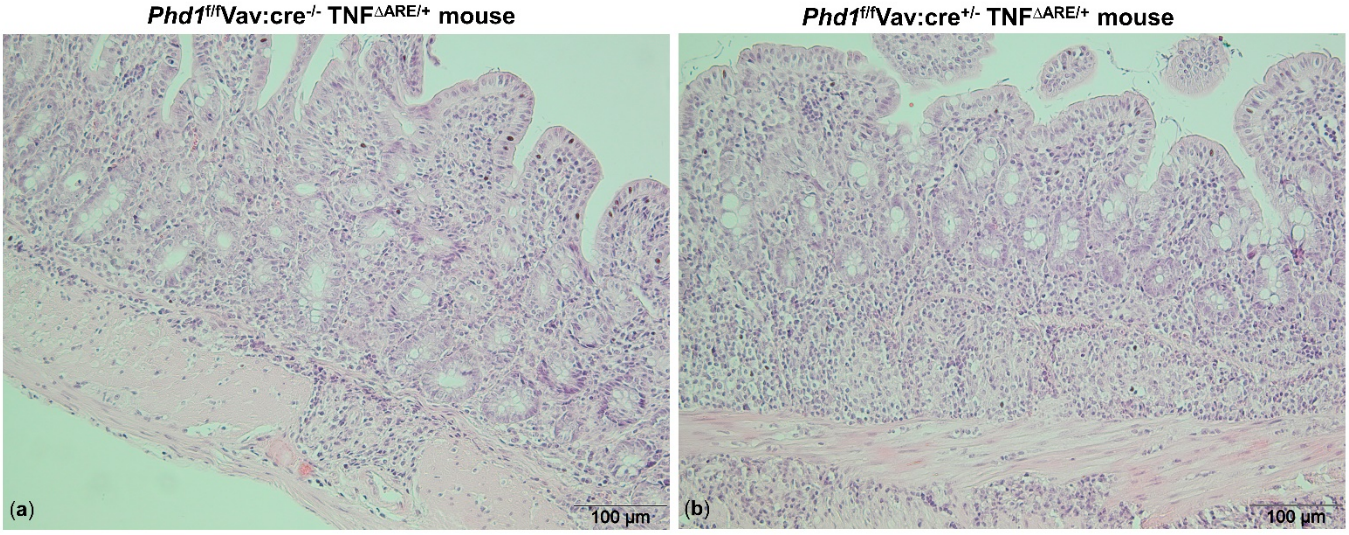

Figure S13. Haematopoietic deletion of Phd1 does not protect against ileitis in TNF$^{\Delta are/+}$ mice. Representative H&E-stained sections from (**a**) a Phd1$^{f/f}$Vav:cre$^{-/-}$ TNF$^{\Delta ARE/+}$ and (**b**) a Phd1$^{f/f}$Vav:cre$^{+/-}$ TNF$^{\Delta ARE/+}$ mouse. .

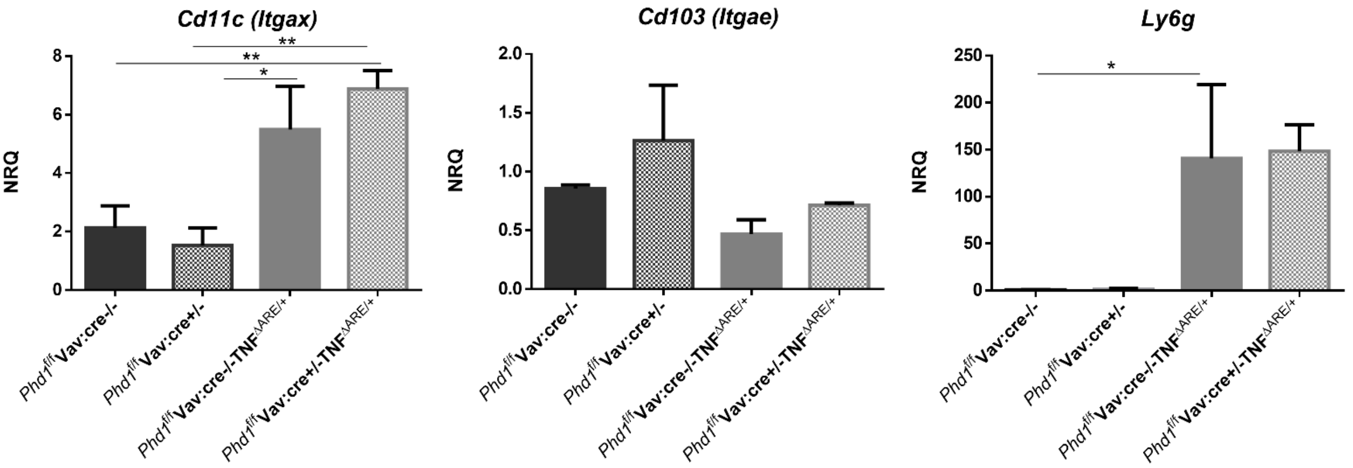

Figure S14. $TNF^{\Delta ARE/+}$ mice, with and without Phd1-deletion, exhibit increased numbers of mononuclear phagocytes. mRNA expression levels of mononuclear phagocytes marker Cd11c (Itgax) (left), the lamina propria dendritic cell-specific marker Cd103 (Itgae) (middle) and the neutrophil marker Ly6g in the distal ileum from $Phd1^{f/f}Vav:cre^{-/-}$, $Phd1^{f/f}Vav:cre^{+/-}$, $Phd1^{f/f}Vav:cre^{-/-}TNF^{\Delta ARE/+}$, $Phd1^{f/f}Vav:cre^{+/-}TNF^{\Delta ARE/+}$. *$p<0.05$; **$p<0.01$. Data are represented as the mean ± SEM. NRQ: Normalized relative quantities.

Table S1. Sequence of used PCR primers.

PCR	Forward primers (5′ - 3′)	Reverse primers (5′ - 3′)	Product size (bp)
Cre	CGCCGTAAATCAATCGATGAGTTGCTTC	GATGCCGGTGAACGTGCAAAACAGGCTC	500
Internal control	CAAACCTGCTACCCGAAGGT	CAGTATGCGGAAGTTCTAGG	800
Phd1 cko	CAGTCCTCCAATAGGCCTCAC	ATTTAGTTCAGTTCTCAGCA	Floxed *Phd1*: 340<br>WT *Phd1*: 290
$TNF^{\Delta ARE}$	CCTTCCTCACAGAGCCAGCCCCCT	AATTACGGTTAGGCTCCTGTTTCC	$TNF^{\Delta ARE}$: 626<br>WT: 560

Legend: Cre: cyclic recombinase ; Phd1: prolyl hydroxylase 1; Phd1 cko: Phd1 conditional knock-out. WT: wild-type

Table S2. Sequence of used qRT-PCR primers.

Gene symbol	Forward primers (5′ - 3′)	Reverse primers (5′ - 3′)
Ccl2	TTAAAAACCTGGATCGGAACCAA	GCATTAGCTTCAGATTTACGGGT
Il1β	CAACCAACAAGTGATATTCTCCATG	GATCCACACTCTCCAGCTGCA
Itgae	CCTGTGCAGCATGTAAAAGAATG	CAAGGATCGGCAGTTCAGATAC
Itgax	CTGGATAGCCTTTCTTCTGCTG	GCACACTGTGTCCGAACTCA
Ly6g	GACTTCCTGCAACACAACTACC	ACAGCATTACCAGTGATCTCAGT
Phd1 ko	AATGGGCGCACCAAGGTACG	GTGATACTGGTACTTGAACACC
Phd1 wt	TCACGTGGACGCAGTAATCC	TAATAGATACAG GTGATGCAGC
Tnf	CATCTTCTCAAAATTCGAGTGACAA	TGGGAGTAGACAAGGTACAACCC
Vegf	ACTCGGATGCCGACACGGGA	CCTGGCCTTGCTTGCTCCCC

Legend: Ccl2: chemokine (C-C motif) ligand 2; Il1β: Interleukin 1 Beta; Itgae (also known as Cd103): Integrin αE; Itgax (also known as Cd11c): Integrin αX; Ly6g: Lymphocyte antigen 6 complex locus G6D; Phd1 ko: Prolyl hydroxylase 1 knock-out allele; Phd1 wt: Prolyl hydroxylase 1 wildtype allele; Tnf: Tumour necrosis factor; Vegf: Vascular endothelial growth factor

SUPPLEMENTARY REFERENCES

1. Van Welden, S.; De Vos, M.; Wielockx, B.; Tavernier, S.J.; Dullaers, M.; Neyt, S.; Descamps, B.; Devisscher, L.; Devriese, S.; Van den Bossche, L.; et al. Haematopoietic prolyl hydroxylase-1 deficiency promotes M2 macrophage polarization and is both necessary and sufficient to protect against experimental colitis. *J. Pathol.* **2017**, *241*, 547–558.
2. Abram, C.L.; Roberge, G.L.; Hu, Y.; Lowell, C.A. Comparative analysis of the efficiency and specificity of myeloid Cre deleting strains using ROSA-EYFP reporter mice. *J. Immunol. Methods* **2014**, *408*, 89–100.
3. Stadtfeld, M.; Graf, T. Assessing the role of hematopoietic plasticity for endothelial and hepatocyte development by non-invasive lineage tracing. *Development* **2005**, *132*, 203-213.
